# The preliminary effect of whole-body vibration intervention on improving the skeletal muscle mass index, physical fitness, and quality of life among older people with sarcopenia

**DOI:** 10.1186/s12877-018-0712-8

**Published:** 2018-01-17

**Authors:** Shu-Fang Chang, Pei-Chen Lin, Rong-Sen Yang, Rea-Jeng Yang

**Affiliations:** 1 School of Nursing, College of Nursing, National Taipei University of Nursing and Health Sciences, 365, Ming Te Road, Pei-Tou, Taipei, 112 Taiwan, Republic of China; 2 Department of Orthopaedics, National Taiwan University & Hospital, No.7, Chung-Shan S. Rd, Taipei, Taiwan, Republic of China; 3 Department of Nursing, National Taipei University of Nursing and Health Sciences, No. 365, Ming Te Rd., Peitou, Taipei City, 112 Taiwan, Republic of China

**Keywords:** Sarcopenia, Quasi-experimental research, Whole-body vibration, Skeletal muscle mass index, Physical fitness, Quality of life

## Abstract

**Background:**

Studies have shown that sarcopenia easily leads to difficulty moving, disability, and poor quality of life. However, researches on the use of whole-body vibration for older adults with sarcopenia living in institutions have been lacking. Therefore, the main objective of the present study was to investigate the effect of whole-body vibration intervention on improving the skeletal muscle mass index, physical fitness, and quality of life of older adults with sarcopenia living in institutions.

**Methods:**

This study adopted a quasi-experimental, single-group, pretest-posttest design. The whole-body vibration intervention was performed over a 3-month period, in which the older adults trained 3 times per week; each training lasted 60 s with a break of 30 s for 10 repetitions. The older adults’ skeletal muscle mass index, physical fitness and quality of life before and after the intervention of the whole-body vibration was collected. Concerning the statistical methods adopted, nonparametric method-based tests were employed.

**Results:**

According to the results of analysis, after the intervention of the 12-week whole-body vibration, the skeletal muscle mass index (z = − 3.621, *p* = 0.000), physical fitness on standing on one foot (z = − 2.447, *p* = 0.014), shoulder–arm flexibility (z = − 3.159, *p* = 0.002), 8-ft up and go test (z = − 2.692, *p* = 0.009), hand grip strength (z = − 3.388, *p* = 0.009), and five repeated sit-to-stand tests (z = − 2.936, *p* = 0.003), all improved significantly. Furthermore, concerning the quality of life of the older adults in the pretest and posttest, the improvements were statistically significant (z = − 2.533, *p* = 0.011).

**Conclusions:**

The study results showed the effect of whole-body vibration intervention on improving the skeletal muscle mass index, physical fitness, and quality of life of sarcopenic older people living in institutions and could serve as a crucial reference to health care professionals.

## Background

Advances in medical technology and improvements in social welfare have increased the human life expectancy. The World Health Organization (WHO) defines ageing society as a society in which at least 7% of its total population is aged 65 years or above, and aged society and super-aged society as a society in which at least 14% and 20% of its population is aged 65 years or above, respectively [1]. As the population of older adults aged 65 years or above continues to grow in Asia. The increase in the average life expectancy of the Taiwanese population and the nation’s annual national average fertility rate continues to decrease; ageing society becomes an irreversible trend.

In 1989, Rosenberg proposed the term “sarcopenia”; in Greek, the prefix “sarx” means “flesh” (i.e., muscles), and the suffix “penia” means “loss.” Sarcopenia is defined as age-related muscle mass reduction [2]. Various studies have used different operational definitions for sarcopenia. The International Working Group on Sarcopenia argued that sarcopenia denotes the combination of muscle loss and a decline in physical performance [3]. The Society on Sarcopenia, Cachexia and Wasting Disorders defined sarcopenia as muscle mass reduction and decreased mobility such as decreased walking speed [4]. The European Working Group on Sarcopenia in Older People described that sarcopenia involves muscle mass reduction coupled with a decline in muscle strength or physical performance; low muscle mass alone is considered pre-sarcopenia, whereas low muscle mass with low physical performance is considered sarcopenia. The combination of low muscle mass, low muscle strength, and low physical performance is considered severe sarcopenia [5]. Beaudart et al. [6] conducted a systematic review and meta-analysis of the health outcomes of sarcopenia. Their results showed high rates of mortality, functional decline, falls, and hospitalizations among older people with sarcopenia.

Studies have shown that the number of older adults aged 65 years or above is approximately 2.6 million in Taiwan, among whom 500,000 have sarcopenia. This signifies that roughly one in every five older adults has sarcopenia [7]. Foreign studies have shown that sarcopenia easily leads to difficulty moving, disability, and poor quality of life [5]. In addition, a meta-analysis showed that the mortality of older adults with sarcopenia is higher than that of older adults without sarcopenia [8]. A study estimated that sarcopenia-led medical expenses total approximately US$11.8–26.2 billion in the U.S. per year [9]. Therefore, a considerable amount of medical costs can be saved annually by effectively preventing sarcopenia.

The use of whole-body vibration intervention to elevate the muscle functions of older adults has gained popularity in recent years. Research has shown that whole-body vibration can enhance the muscle strength and functional movement efficiency of older adults, prevent them from falling, and improve their health and quality of life [10]. Other studies have shown that whole-body vibration can improve muscle functions in a manner similar to that of traditional resistance training. However, compared with resistance training, whole-body vibration is safer and more convenient and avoids older adults from the risk of resistance training-led injuries [11].

Whole-body vibration therapy is of three types: vertical-sinusoidal whole-body vibration (VS-WBV), side-alternating whole-body vibration (SS-WBV), and stochastic-vertical whole body vibration (SR-WBV). Users are asked to sit or stand on a VS-WBV or SS-WBV device, in which a high frequency of 20–50 Hz and an amplitude of 2–14 mm are generally used. For SR-WBV devices, a frequency of 1–12 Hz and amplitude of 3–6 mm are generally used. In these devices, users are asked to place their feet on two independently powered vibration platforms [12–14]. Whole-body vibration is safe and effective in elevating the muscle strength of older adults, while being widely adopted on older adults living in communities and menopausal women as a physical therapy measure. However, studies on the use of whole-body vibration for older adults with sarcopenia living in institutions have been lacking. The present study is thus the first-ever interventional study that investigated the use of whole-body vibration in sarcopenic older people, marking its innovativeness and importance. Therefore, the study results may serve as a crucial reference to health care professionals in caring for older adults with sarcopenia.

### Aims

The present study first investigated the demographics, skeletal muscle mass index (SMMI), physical fitness, and quality of life of sarcopenic older people living in institutions. Next, the study explored the longitudinal effect of whole-body vibration on the sarcopenic older people’s SMMI, physical fitness, and quality of life.

### The research question

Can whole-body vibration training improve the SMMI, physical fitness, and quality of life of older people with sarcopenia?

## Methods

In this study, a longitudinal, quasi-experimental, single-group pretest-posttest design was adopted without allocation concealment procedure to examine the effect of whole-body vibration on improving older people’s SMMI, physical fitness, and quality of life.

### Study participants

The inclusion criteria were participants aged older than 65 years who lived in nursing homes and care centers in Taipei, Taiwan. Because the Asian Working Group for Sarcopenia (AWGS) adopts a more stringent standard for diagnosing sarcopenia among older people [15], patients with AWGS-diagnosed sarcopenia are generally wheelchair- or hospital bed-bound. Therefore, they are unable to participate in the standing-based whole-body vibration intervention experiment conducted in this study. Thus, in this study, Asian older people with sarcopenia were selected according to the diagnostic standard proposed by Shahar et al. [16]. Standards proposed by Shahar et al. [16] were used to identify participants with sarcopenia; such standards included males with a grip strength of less than 26 kg, females with a grip strength of less than 18 kg, males and females with a walking speed of less than 0.8 m/s, males with SMMI of less than 10.75 kg/m^2^, and females with an SMMI of less than 6.75 kg/m^2^. The participants were selected using the purposive sampling method, in which the participants had to be at least 60 years of age, were clearly conscious, could communicate in Mandarin or Taiwanese, and scored ≥21 on the mini–mental state examination. The study objectives were then explained to the participants, and those who were willing to participate in this study and signed the consent form were recruited.

The participant exclusion criteria included participants with an acute injury, a bone fracture, hip and/or thigh joint damage, a surgery within the past month, a severe heart disease (that involved cardiac stent implantation or a bypass surgery), a coronary artery disease, a pacemaker, a serious mental or cognitive disorder, a score of < 21 on the mini–mental state examination, and/or a severe visual or hearing problem.

### Intervention protocol

This study adopted a quasi-experimental one-group pretest–posttest design and followed recommendations by Chen et al. [15]. The i-vib6050 model was selected. The participants were asked to stand on a vibration and stimulation-generating platform with a vibration frequency and amplitude of 12 Hz and 3 mm, respectively. During the 3-month study period, three sessions were performed each week. Each session comprised 10 repetitions, each lasting 60 s, followed by a 30-s resting period. For each session, research personnel conducted whole-body vibration training on the devices at participants’ wards at nursing homes or care centers. The at-ward practice of this intervention prevented the increased physical activity resulting from participants’ traveling from ward to the study center, which may become a potential confounding factor affecting the study results. Before and after the intervention, the variables of SMMI, physical fitness, and quality of life of older adults with sarcopenia were collected.

### Demographic information

The participants’ demographic information included their gender, age, height, educational attainment, medical history, previously experienced fall, and frailty assessment.

### Skeletal muscle mass index (SMMI)

SMMI was obtained using a body composition analyzer. SMMI was calculated using the following formula: total skeletal muscle mass (in kg)/body weight (in kg) × 100. This study used an improved body composition analyzer (model IOI353) to measure participants’ body muscle mass. Because the inclusion criteria in this study were adults aged older than 65 years who lived in long-term care facilities, we did not ask them to fast because of health and safety reasons. Instead, we measured their SMMI 3 h after they ate.

The device weighed approximately 20 kg, contained portable wheels and a handle, and was easy to carry. Thus, it can be used by older people living in communities or institutions. In addition, because the appearance of the device is comparable to that of scales used in hospitals, it can be conveniently placed in nursing stations. Compared with traditional bioelectrical impedance analysis (BIA) instruments, which can only evaluate the weight of subcutaneous fat and not the weight of visceral fat, the IOS353 can accurately measure body muscle mass and the weight of visceral fat. In addition, it has a CT accuracy correlation of 0.902 and a dual energy X-ray absorptiometry (DEXA) accuracy of 0.97 [17].

### Physical fitness

The various functional fitness of the participants were assessed using the senior physical fitness test proposed by Rikli and Jones [18]. The test items are listed as follows: standing on one foot, an upper arm flexibility–arm curl test (hands locking each other behind the back), an 8-ft up and go test, a grip strength test, a “five repeated sit-to-stand” test and. The physical fitness test, designed to effectively evaluate elderly adults’ abilities to physically perform daily living activities, is commonly used to evaluate their functional fitness [18, 19]. According to the study objectives, the present study also added the standing on one foot test to measure the elderly adults’ ability to balance while in a still position.

#### Standing on one foot

The elderly adults were asked to stand on one foot while bending the knee of the other leg by approximately 90°. Next, the amount of time (measured in seconds) that the elderly adults maintained their balance was measured. In a related study, Franchignoni et al. [20] conducted a standing on one foot test on 45 women over the age of 55, where the results showed that the test had an intraclass correlation coefficient (ICC) and content validity of 0.98 and 0.83, respectively.

#### Shoulder–arm flexibility

The elderly adults were asked to lift one of their arms above their shoulders and then move it downwards behind their back. Next, they were asked to move the other arm upwards from their waist behind their back. The distance between the two middle fingers was then measured. The same procedure was repeated by alternating the arms and the better score out of the two tests was selected as the elderly adults’ upper limb flexibility. The intraclass correlation coefficient and content validity of this test were 0.83 and 0.82, respectively [18].

#### Eight-foot up and go test

The present study used the 8-ft up and go test to measure the elderly adults’ ability to maintain their balance while moving. For the test, the elderly adults were asked to sit in a chair, stand up and walk for eight feet at the sound of the signal, bypass the flag pole, and return to their seat and sit down. The amount of time that the elderly adults needed to complete the tasks were measured, which was used to evaluate their agility and ability to maintain their balance while moving. The intraclass correlation coefficient and content validity of this test were 0.98 and 0.85, respectively [18].

#### Grip strength

In this study, a JAMAR hydraulic grip strength device (model J00105; Lafayette, USA) was used to measure the maximum muscle strength of participants’ upper limbs during static contraction. To assess test–retest reliability, the grip strength device was subsequently used to measure the muscle strength of both hands of 21 healthy older people. No considerable differences were observed between the measured results of the participants and healthy older people, and test–retest reliability for left and right hands was 0.954 and 0.912, respectively. In addition, the intraclass correlation coefficient this test was 0.81 and the content validity of this test was 0.81 and 0.78 for the male and female elderly adults [21], respectively.

This study followed validated standardized guidelines for grip measurement [22]. The present study used an electronic grip strength device to measure the maximum muscle strength of the elderly adults’ upper limbs. For the test, the elderly adults were asked to squeeze three times on the aforementioned device with their left or right hand. Next, they were asked to switch hand and squeeze the device once again; the same procedure was repeated once, and their muscle strengths were measured. The highest grip strength displayed across six trials was selected as the elderly adults’ grip strength. This test enabled an evaluation of the elderly adults’ upper limb muscle performance.

#### Five repeated sit-to-stand test

The present study used the chair stand test to measure the muscle fitness performance of the elderly adults [18]. The elderly adults were asked to have their arms crossed in front of their chest and stand up from a sitting position five straight times; the amount of time (measured in seconds) that the elderly adults needed to complete the task was measured. This test allowed an evaluation of the elderly adults’ lower limb muscle performance. In a functional fitness-related study, Rikli and Jones [18] administered a five repeated sit-to-stand test on 82 elderly adults aged 65 years or above and who were living in communities, where the results showed that the test had an intraclass correlation coefficient and content validity of 0.95 and 0.83, respectively.

### Quality of life

The EQ-5D-5 L quality of life questionnaire consists of two parts, which are a descriptive system and a visual analog scale. For the descriptive system, respondents evaluate their health status in terms of five dimensions (i.e., mobility, self-care, usual activities, pain/discomfort, and anxiety/depression). For the visual analog scale (VAS), respondents rate their health on a scale of 0 to 100, in which 0 and 100 correspond to the worst and best health that the respondent can imagine, respectively. The respondent is then required to draw a line from the box on the questionnaire to the scale to indicate his/her health state on the interviewed day. The EQ-5D-5 L quality of life questionnaire showed reliability, validity, and diagnose accuracy rate of 0.82, 0.80, and 92%, respectively [23, 24].

### Sample size

The G-Power 3.1 software program was used to calculate the sample size. The α level, power, and effect size were set as 0.05, 0.80, and 0.4, respectively, which produced a minimum sample size of 26. The number of participants who met the inclusion criteria and who were willing to participate in this study totaled 31 during the pretest. However, because some participants were discharged from the institution, experienced poor physical condition(s), and/or withdrew from the study during the study; only 17 participants remained during the posttest. Therefore, measurement variables were calculated (via software G-POWER, model 3.1.9) using the means and standard deviations of the dependent variables, in which the Power and alpha level were set at 0.95 and 0.05, respectively. According to the results, all of the effects were at 0.4 or above, indicating that all of the measurement variables featured favorable effects (Table 1).

**Table 1:** Test power and effect size of measured variables obtained using various variables

Measured variables	Pretest (*n* = 17)	Posttest (*n* = 17)	Test power	Effect size
	Mean ± SD	Mean ± SD		
SMMI	8.97 ± 1.69	10.52 ± 2.35	0.95	0.7529
Physical fitness				
Standing on one foot (seconds)	3.30 ± 7.19	7.36 ± 12.32	0.95	0.4023
Shoulder–arm flexibility (centimeters)	−37.50 ± 16.33	− 28.56 ± 13.94	0.95	0.5884
8-ft up and go test (seconds)	36.2 ± 24.57	22.00 ± 17.25	0.95	−0.7091
Grip strength (kilograms)	11.19 ± 7.36	14.45 ± 7.48	0.95	0.4399
Five repeated sit-to-stand tests (seconds)	42.42 ± 22.98	30.58 ± 15.31	0.95	−0.6063

*SMMI* Skeletal muscle mass index

### Ethical considerations

This study passed the review and ethical approval by the Behavioral and Social Sciences Research Ethics Office of National Taiwan University (IRB-Reference Code: 201402ES013) in Taiwan. To protect the privacy as well as the rights and interests of the study participants, the study objectives, methods, and amount of time to be spent were explained to the older people. The older people could decide whether to take part in the study or withdraw midway without it having an adverse effect on the care that they received in the institution. All the written consent was obtained from participants. The research data were solely used for research purposes and strictly kept confidential. The names of the participants and the questionnaire numbers were replaced with codes prior to analyses to ensure the participants’ data security.

### Statistical analysis

After coding and decoding the collected data, the present study performed data analyses by using the statistical analysis software of SPSS for windows 20.0 (Chinese version). Percent, mean, and standard deviation were employed. The Kolmogoron-SMMInov (normal test) was adopted to explore the normal distribution situation of the sample during the pretest, where the results showed that the sample exhibited a nonnormal distribution (*p* < .05). Accordingly, nonparametric method-based tests with the Wilcoxon test for pretest and posttest.

## Results

### The demographics, SMMI, physical fitness, and quality of life among older people

The average height and age of the participants were 159.71 ± 9.51 cm and 82.12 ± 8.19, respectively. Of the 17 participants recruited in this study, the majority of them were male (70.6%). Among the participants, 71.6% had an educational attainment of elementary school or higher. The medical history of the participants showed that those with diabetes accounted for the highest proportion (23.5%). Roughly 52.9% of the participants had previously experienced fall(s). The SOF index showed that most of the participants were in the prefrail stage (Table 2).

**Table 2:** The demographics of sarcopenic older people living in institutions and SMMI, physical fitness, and quality of life among older adults with sarcopenia (*N* = 17)

Variables	Mean ± SD	n (%)
Height	159.71 ± 9.51	
Age	82.12 ± 8.19	
Gender		
Male		12(70.6)
Female		5(29.4)
Education Level		
Illiterate		5(29.4)
Literate		1(5.9)
Elementary school		3(17.6)
Junior high school		1(5.9)
College		2(11.8)
University or higher		5(29.4)
Diseases (may select more than one)		
Stroke		2(11.8)
Cardiovascular disease		3(17.6)
Diabetes		4(23.5)
Underwent recent surgery		1(5.9)
Previously experienced fall(s)		
Yes		9(52.9)
No		8(47.1)
Frailty assessment (SOF index)		
Normal		2(11.8)
Prefrail stage		15(88.2)
Frail stage		–
SMMI	8.98 ± 1.69	
Physical fitness		
Standing on one foot (seconds)	3.30 ± 7.19	
Shoulder–arm flexibility (centimeters)	−37.50 ± 16.33	
8-ft up and go test (seconds)	36.2 ± 24.57	
Grip strength (kilograms)	11.19 ± 7.36	
Five repeated sit-to-stand tests (seconds)	42.42 ± 22.98	
Quality of life		
Index	0.69 ± 0.24	
VAS	78.24 ± 8.28	

*SMMI* Skeletal muscle mass index, *VAS* visual analog scale

The average SMMI was 8.97 (SD = 1.69) for the participants. Concerning the physical fitness, the average results of the various tests that the participants underwent are listed as follows: (a) standing on one foot was 3.30s (SD = 7.19); (b) shoulder–arm flexibility was − 37.50 cm (SD = 16.34); (c) 8-ft up and go test was 36.20 s (SD = 24.57); (d) hand grip strength was 11.19 kg (SD = 7.36); and (d) five repeated sit-to-stand test was 42.42 (SD = 22.98) times. Regarding the quality of life of the participants, on average, the participants scored 0.69 (SD = 0.24) for their health-related quality of life index and 78.24 (SD = 8.28) for visual analog scale (VAS) (Table 2).

### The longitudinal effect of whole-body vibration on SMMI, physical fitness, and quality of life

The present study used the Wilcoxon test to analyze the SMMI, physical fitness, and quality of life before and after the intervention of whole-body vibration. According to the results, significant differences were observed in the SMMI (z = − 3.62, *p* < .05), standing on one foot test (z = − 2.45, *p* < .05), Shoulder–arm flexibility (z = − 3.16, *p < .05*), 8-ft up and go test (z = − 2.69, *p* < .05), hand grip strength (z = − 3.39, *p* < .05), time to complete five repeated sit-to-stand test (z = − 2.94, *p* < .05), and quality of life index (z = − 2.53, *p* < .05) (Table 3).

**Table 3:** The effect of whole-body vibration on the SMMI, physical fitness, and quality of life (*n* = 17)

Variables	Pretest MEAN ± SD	Posttest MEAN ± SD	z	*p* value
SMMI	8.98 ± 1.69	10.63 ± 2.24	−3.62	0.000***
Physical fitness				
Standing on one foot (seconds)	3.30 ± 7.19	7.37 ± 12.32	−2.45	0.014**
Shoulder–arm flexibility (centimeters)	−37.50 ± 16.33	−28.56 ± 13.95	−3.16	0.002**
8-foot up and go test (seconds)	36.2 ± 24.57	22.00 ± 17.36	−2.69	0.009**
Grip strength (kilograms)	11.19 ± 7.36	14.46 ± 7.48	−3.39	0.001**
Five repeated sit-to-stand tests (seconds)	42.42 ± 22.98	30.58 ± 15.32	−2.94	0.003**
Quality of life				
Index	0.69 ± 0.24	0.74 ± 0.23	−2.533	0.011*
VAS	78.24 ± 8.28	80.29 ± 10.68	−0.672	0.501

*SMMI* Skeletal muscle mass index, *VAS* visual analog scale; *p < .05, ** p < .01, *** p < .001

## Discussion

This study first explored the demographics, physical fitness, and quality of life of sarcopenic older persons living in institutions. Secondly, the research studied the longitudinal effect of whole-body vibration on the sarcopenic older persons’ SMMI, physical fitness, and quality of life. According to the results of this research, after the intervention of the 12-week whole-body vibration, the SMMI and physical fitness improved significantly, respectively. Meanwhile, the quality of life of the older people in the pretest and posttest, the improvements were also founded.

### Demographics, SMMI, physical fitness, and quality of life among sarcopenic older people

Adults aged older than 65 who lived in nursing homes and had sarcopenia were included in this study. The results showed that their average age was 82 years, indicating that sarcopenia is more common among older adults. Chang and Lin [8] conducted a systematic literature review and meta-analysis; they determined that sarcopenia is common among older adults and that the risk of death is higher for older adults with sarcopenia than for those without sarcopenia. Meanwhile, this study showed that male older people outnumbered female older people, which matched the results of Wu et al. [25] and Chien, Huang, and Wu [26]. In addition, the study revealed that concerning the different diseases that the older adults had, the most common was diabetes, followed by hypertension. Kim et al. [27] studied 145 older adults (aged 65 years or above) with sarcopenia and found that 73% and 56% of them had diabetes and hypertension, respectively. Landi et al. [28] studied 40 older adults (aged 70 years or above) with sarcopenia and who lived in nursing homes, in which they found that 66% and 18% of them had hypertension and diabetes, respectively. Ishii et al. [29] conducted a muscle mass study and studied older adults with sarcopenia and who lived in communities, in which they found that 48.5% and 13% of the older adults had hypertension and diabetes, respectively. These studies showed that older adults with sarcopenia experience chronic diseases and that comorbidity regularly exists.

In the present study, more than half of the older people had previously experienced fall(s), which supported the results obtained from related studies (i.e., older adults with sarcopenia have a higher risk of falling). Yamada et al. [30] studied 414 older adults with sarcopenia and found that they were afraid of falling and had previously experienced fall(s), and that fall(s) is a sarcopenia-related factor. The frailty assessment of the present study showed that 88.2% of the older people were in the prefrail stage. According to Morley et al. [31], sarcopenia reduces muscle strength and walking speed, which decrease mobility and overall calorie consumption as well as increase the severity of frailty. Scholars have pointed out that sarcopenia is a manifestation of frailty and that both sarcopenia and frailty are related to musculoskeletal system ageing [32].

Concerning the physical fitness in the present study, the results matched the results obtained from related studies. In the study of 414 older adults with sarcopenia conducted by Yamada et al. [30], the male and female participants had a walking speed of ≤1.0 m/s and < 0.8 m/s, respectively; the female participants outperformed the male participants in terms of their flexibility; and the male and female participants had a mean grip strength of 22 kg and 15 kg, respectively. Lee et al. [33] conducted a sarcopenia-related study on older adults over the age of 65 and living in Yilan’s communities, and found that the male and female participants displayed a mean torso muscle mass of 22.6 ± 3.0 and 16.0 ± 2.2, respectively, and a mean grip strength of > 26 kg and > 18 kg, respectively; both the male and female participants had a walking speed of > 1.0 m/s. These studies showed that the participants of the present study had worse physical fitness than older adults with sarcopenia and living in communities did, which may be because of the differences in the environments between institutions and communities, and that the older people lacked regular exercises and physical activities.

Although EQ-5D-5 L, consists of a descriptive system and a visual analog scale (VAS), and it has been proven to demonstrate high reliability and validity when used to assess all patient types including those with stroke, sarcopenia, cardiovascular diseases, respiratory diseases, depression, diabetes, liver diseases, personality disorders, and arthritis [34–38]. However, in 2015, Beaudart et al. developed a sarcopenia-specific quality of life questionnaire (the SarQoL) [39]. In 2017, they tested the psychometric properties (i.e., reliability and validity) of the research instruments, and the results showed that the research instruments exhibited favorable discriminative power, high internal consistency, consistent construct validity, and excellent test–retest reliability [40, 41]. Therefore, they recommended that the SarQoL questionnaire should be used to assess the quality of life of older adults with sarcopenia.

### The longitudinal effect of whole-body vibration on SMMI, physical fitness, and quality of life

After 12 weeks of whole-body vibration, the SMMI became significantly higher than those before the intervention of whole-body vibration. These results supported the finding obtained from related studies. Machado, López, Gallego and Garatachea [42] introduced a 10-week-long whole-body vibration to older adults with sarcopenia, in which the result showed that the experimental group participants’ muscle mass increased significantly after the 10-week-long whole-body vibration. Bogaerts et al. [43] and Verschueren et al. [44] found that whole-body vibration can improve bone density, enhance muscle quality, and lower body fat. However, some studies showed that whole-body vibration did not significantly improve the muscle mass of older adults with sarcopenia [45–47]. The results of these studies differed from those of the present study because the vibration amplitude, vibration frequency, rest interval, and intervention duration varied across the studies.

In this study, after the12-week-long whole-body vibration intervention, the older people showed significant improvements in their physical fitness. These results matched that obtained from related studies. Wei et al. [46] introduced a 12-week-long whole-body vibration to older adults with sarcopenia, where the results showed that the older adults’ five repeated sit-to-stand time and 10 m walking speed improved significantly after the intervention of the whole-body vibration. Delecluse, Roelants, and Verschueren [48] indicated that whole-body vibration creates mechanical vibrations and promotes the synergist and antagonist muscle effect. Wu, Chen, and Chen [49] studied the effect of a 6-week-long whole-body vibration and stretching on the functional fitness of 30 female older adults. Result indicates that whole-body vibration and stretching can significantly improve female older adults’ lower limb muscle strength, agility, balance while moving, and flexibility. Kawanabe et al. [50] provided 67 older adults with a 2-month-long whole-body vibration, where they found that the whole-body vibration group’s walking pace and standing on one foot time increased significantly, and that the amount of time for them to walk 10 m decreased significantly. Bosco et al. [51] examined the effect of whole-body vibration on improving older adults’ body composition and physical fitness, in which the results showed that it significantly improved their lower limb muscle strength and balance.

Regarding the older persons’ healthy quality of life index, it increased significantly after the intervention of the 12-week-long whole-body vibration. This result matched that of related studies. Álvarez-Barbosa et al. [52] investigated the risk of falling, daily living activities, and healthy quality of life of 29 older adults living in an institution after the intervention of an 8-week-long whole-body vibration. The results showed statistically significant differences in the EQ-5D-5 L healthy quality of life index and the visual analog scale between the pretest and posttest. Studies that recruited different test populations also showed that whole-body vibration can improve the EQ-5D-5 L index. Baumbach et al. [53] introduced a 6-week-long whole-body vibration to 60 participants. The participants’ mean EQ-5D-5 L index in the posttest was significantly higher than that of the pretest.

### Limitations

The present study is the first-ever study to investigate the effect of whole-body vibration on improving the SMMI, physical fitness, and quality of life of sarcopenic older adults living in long-term care facilities, thereby marking the innovativeness and importance of this study. Nevertheless, this study still encountered a few limitations: First, this study conducted quasi-experimental, only single-group, pretest-posttest designs. Therefore, research can not reach allocation concealment procedure. Meanwhile, because this study was conducted in a quasi-experimental design, the external validity had limited explanatory power to make inferences. Second, because of safety reasons, we did not ask the older adults to fast. Instead, we measured their SMMI 3 h after they ate; hence, food consumption by the participants might have influenced the SMMI measurement. Third, this study adopted a quasi-experimental, single-group pretest-posttest design method, which better matched the real life situations of natural studies and featured less variable controls. However, such a method lacked a control group to enable a comparison between participants who underwent whole-body vibration and those who did not. Finally, this study required the participants to spend a relatively long time (i.e., an average of 30–40 min) to complete the study tools, which may have affected the participants’ willingness to complete them as well as the credibility of the results. Therefore, future studies may consider simplifying the study tools or providing the participants with a place to rest during repeated assessments to ensure that the study data better match actual experiment results.

## Conclusions

This study showed that the intervention of the 12-week-long whole-body vibration enhanced the physical fitness and quality of life among sarcopenic older persons who lived in institutions. The study results may serve as crucial references to health care professionals in performing whole-body vibration intervention to sarcopenia older persons. In addition, this study recommends that health care professionals need to provide sarcopenia older adults living in long-term care facilities with whole-body vibration as well as regularly monitor the effect of whole-body vibration on their SMMI, physical fitness, and quality of life.

## Abbreviations

BIA: Bioelectrical Impedance Analysis
DEXA: Dual Energy X-ray Absorptiometry
EQ-5D-5 L: EuroQoL 5 Dimensions 5 Levels
SMMI: Skeletal Muscle Mass Index
SR-WBV: Stochastic-Vertical Whole Body Vibration
SS-WBV: Side-alternating Whole Body Vibration
VAS: Visual Analog Scale
VS-WBV: Vertical-Sinusoidal Whole Body Vibration
WBV: Whole-body vibration
